# Evaluation of an antibody-PNA conjugate as a clearing agent for antibody-based PNA-mediated radionuclide pretargeting

**DOI:** 10.1038/s41598-020-77523-y

**Published:** 2020-11-27

**Authors:** Anders Myrhammar, Anzhelika Vorobyeva, Kristina Westerlund, Shuichiro Yoneoka, Anna Orlova, Takehiko Tsukahara, Vladimir Tolmachev, Amelie Eriksson Karlström, Mohamed Altai

**Affiliations:** 1 Department of Protein Science, School of Engineering Sciences in Chemistry, Biotechnology and Health, KTH Royal Institute of Technology, Stockholm, Sweden; 2 Department of Immunology, Genetics and Pathology, Uppsala University, Uppsala, Sweden; 3 Laboratory for Advanced Nuclear Energy, Tokyo Institute of Technology, Tokyo, Japan; 4 Department of Medicinal Chemistry, Uppsala University, Uppsala, Sweden; 5 Research Centrum for Oncotheranostics, Research School of Chemistry and Applied Biomedical Sciences, Tomsk Polytechnic University, Tomsk, Russia; 6 Division of Oncology and Pathology, Kamprad Lab, Department of Clinical Sciences, Lund University, Lund, Sweden

**Keywords:** Biochemistry, Biotechnology, Chemical biology, Molecular medicine, Oncology

## Abstract

Radionuclide molecular imaging of cancer-specific targets is a promising method to identify patients for targeted antibody therapy. Radiolabeled full-length antibodies however suffer from slow clearance, resulting in high background radiation. To overcome this problem, a pretargeting system based on complementary peptide nucleic acid (PNA) probes has been investigated. The pretargeting relies on sequential injections of primary, PNA-tagged antibody and secondary, radiolabeled PNA probe, which are separated in time, to allow for clearance of non-bound primary agent. We now suggest to include a clearing agent (CA), designed for removal of primary tumor-targeting agent from the blood. The CA is based on the antibody cetuximab, which was conjugated to PNA and lactosaminated by reductive amination to improve hepatic clearance. The CA was evaluated in combination with PNA-labelled trastuzumab, T-Z*HP1,* for radionuclide HER2 pretargeting. Biodistribution studies in normal mice demonstrated that the CA cleared ca. 7 times more rapidly from blood than unmodified cetuximab. Injection of the CA 6 h post injection of the radiolabeled primary agent [^131^I]I-T-*ZHP1* gave a moderate reduction of the radioactivity concentration in the blood after 1 h from 8.5 ± 1.8 to 6.0 ± 0.4%ID/g. These proof-of-principle results could guide future development of a more efficient CA.

## Introduction

The use of monoclonal antibodies (mAbs) recognizing cancer-specific molecular abnormalities is one of the more promising ways to treat disseminated cancer. However, early patient stratification is necessary to identify subgroups of patients with tumors expressing a particular antigen and who would respond to targeted therapy. Cancers are very heterogeneous diseases, and tumors of the same origin might have different antigen expression or not express the antigen at all. Moreover, there might be an appreciable intra- and inter-tumor heterogeneity of a target expression within the same patient. Additionally, the expression level might be changed during course of a disease or in response to therapy. This necessitates regular determination of expression level at different tumor sites within the same patient. Such necessity is not compatible with the current method used in clinical practice, biopsy sampling. It has been suggested that the use of radionuclide molecular imaging would be a better alternative to biopsy-based methods, because the imaging is non-invasive and can be performed repeatedly during the treatment course.

The most commonly used approach to molecular imaging is the labeling of therapeutic antibodies with positron-emitting nuclides (immunoPET). An accumulation of such antibodies in tumor sites can be visualized when a target is sufficiently expressed. A drawback of using bulky antibodies are their slow clearance from blood, which results in high background, low imaging contrast and poor sensitivity. To overcome this, a pretargeting approach has been suggested. In pretargeting, a tumor-targeting antibody is conjugated (or fused) with a recognition tag. Such a construct is called a primary agent. After localization of the primary agent in tumors and clearance of the construct from blood, a radiolabeled secondary agent is injected. The secondary agent is designed to have specific and high affinity binding to the recognition tag in the primary agent, and a rapid clearance from blood.

The use of peptide-nucleic acids (PNA) as a recognition mechanism for pretargeting was proposed by the group of Donald Hnatowich in the 1990s but was then forgotten for many years^1^. We have re-vitalized PNA-based pretargeting to solve the problem of high renal uptake during affibody-based radionuclide therapy. Two complementary PNA-based hybridization probes, *HP1* and *HP2,* have been developed^2^. The primary probe *HP1* has been coupled to a targeting affibody molecule by a sortase A-catalyzed ligation and the secondary probe *HP2* has been labelled using radiometals and radiohalogen^2^. *HP1* and *HP2* have demonstrated strong and specific hybridization in vitro, and the feasibility of affibody-based PNA-mediated pretargeting in vivo was demonstrated in an imaging study^3^. These probes have also successfully been used in an experimental affibody-based pretargeted therapy in mice^4^. The Affibody scaffold was developed from the Z domain of protein A, which has a natural affinity to the Fc region of IgG^5^. By enzymatically attaching the PNA-based probe to a photoactivatable Z domain, site-specific coupling of *HP1* to the monoclonal antibody trastuzumab was demonstrated^6^. The use of covalent photoconjugation ensured that the binding of Z*HP1* to the antibody was irreversible^7^. This trastuzumab-Z*HP1* construct has been used together with ^57^Co-labeled *HP2* in a proof-of-concept pretargeting imaging study^6^. This approach has the potential for rapid conversion of any therapeutic antibody into a pretargeting primary probe for companion diagnostics and patient stratification. An advantage of such an approach is the well-defined composition and structure of the primary probe, ensuring a reproducible biodistribution. Still, the clearance time of such an antibody-PNA construct from blood is slow, and optimal imaging was only possible 48 h after injection^6^.

**Figure 1:**
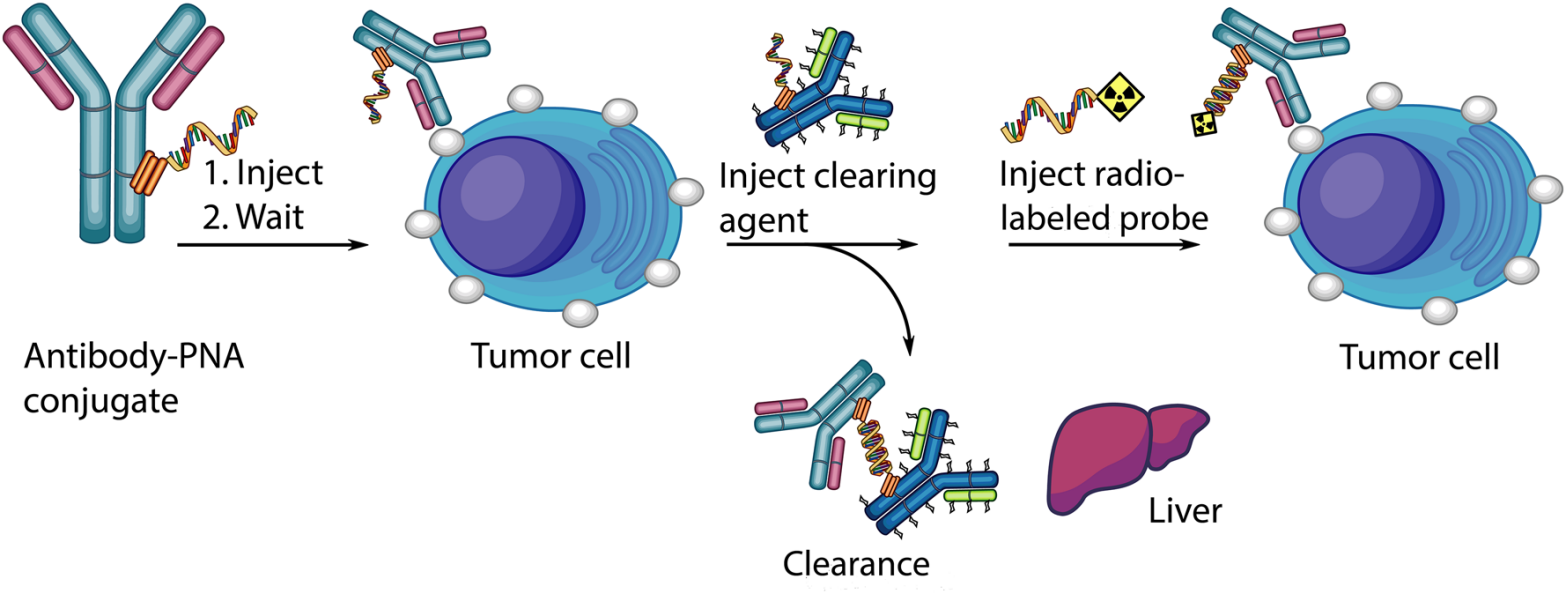
Schematic figure showing the clearing agent concept.

Other studies suggest that the time interval between injections of primary and secondary probes might be appreciably reduced by the use of a clearing agent (CA)^8–10^. The CA should specifically bind the primary agent in circulation and remove it from blood, but not prevent the binding of the secondary probe to the primary agent localized in tumors (Fig. 1). For this purpose, the CA should meet the following requirements: it should specifically recognize the primary probe, efficiently remove the primary probe-CA complex from blood, have inefficient penetration of the CA into tumors to prevent interference with binding of the secondary probe to the recognition tag, and have a favorable safety profile.

In this study, we evaluated if a therapeutic antibody coupled with a complementary PNA, via the Z-domain, might be used as a CA in antibody-based PNA-mediated pretargeting. In our opinion, this approach has several potential advantages. First, the use of an approved therapeutic antibody eliminates the question of safety. Second, the decoration of the CA with Z-PNA enables a modular design of the whole pretargeting system permitting its assembly from a few common components. Third, a CA based on a full-length IgG would extravasate slowly, which minimizes the risk of blocking the antibody-PNA primary agent localized at the tumor site.

**Figure 2:**
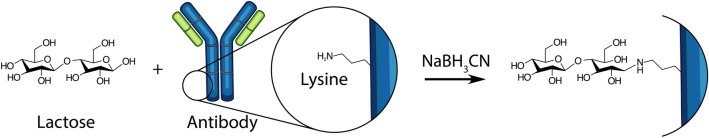
Lactosamination by reductive amination of antibody lysine side chains.

To make the elimination of the primary probe/clearing agent complex more efficient we proposed a lactosamination of our PNA-labeled mAb-based clearing agent (Fig. 2). Lactosamination represents a simple, rapid and mild procedure to increase plasma clearance and catabolism of protein-based agents through binding to asialoglycoprotein receptors (ASGPRs)^11^. ASGPRs are primarily expressed in liver hepatocytes at high levels (500,000 per cell) and are only weakly expressed elsewhere. Binding of ligands to ASGPRs is generally followed by rapid internalization of the ASGPR-ligand complex and vesicle transport to lysosomes for ligand degradation^12^. The receptor’s natural ligands are desialylated glycoproteins with exposed galactose- or *N*-acetylgalactosamine-terminated polysaccharides, and numerous artificially galactosylated and lactosaminationed proteins have also been reported to be efficiently taken up and catabolized in the liver through ASGPR-mediated endocytosis^13–16^. Lactosamination of mAbs and IgG Fab fragments, directed towards angiotensin-converting enzyme in lung and low density lipoprotein in blood, resulted in enhanced liver uptake, rapid blood clearance, and a several-fold increase in the target-to-nontarget uptake ratio for these targeting agents^17,18^. The aim of this current proof-of-principle study is to evaluate a lactosaminated and PNA-conjugated mAb as a clearing agent for the removal of non-bound and circulating PNA-based pretargeting primary agent from blood. The CA is designed to hybridize with high specificity to the primary agent through PNA/PNA recognition and to clear the resulting CA-primary agent complex through ASGR-mediated endocytosis in liver hepatocytes.

## Methods

The majority of the chemicals used in the study were purchased from Sigma-Aldrich Sweden AB. All buffers were prepared using high quality Milli-Q water (resistance higher than 18MΩ cm). Iodide isotopes [^125^I]NaI and [^131^I]NaI were purchased from PerkinElmer Sverige AB (Upplands Väsby, Sweden). Instant thin-layer chromatography (iTLC) analysis was performed using iTLC silica gel strips (Varian Medical Systems, Palo Alto, CA, USA). The activity distribution was measured using a Cyclone storage phosphor system and analyzed using OptiQuant image analysis software (version 2.5) (both from PerkinElmer, Waltham, MA, USA). Size-exclusion chromatography was performed using NAP-5 columns (GE Healthcare, UK). Activity was measured using an automated gamma-spectrometer with NaI(TI) detector (1480 Wizard; PerkinElmer Wallac Oy, Turku, Finland). SKOV-3 cells were purchased from the American Type Culture Collection and were cultured in RPMI-1640 medium (Biochrom GmbH, Berlin, Germany) supplemented with 10% fetal bovine serum (Sigma-Aldrich; Merck KGaA, Darmstadt, Germany), 2 mM l-glutamine, 100 IU/ml penicillin and 100 µg/ml streptomycin in a humidified incubator with 5% CO_2_ at 37 ˚C, unless stated otherwise.

### Synthesis and purification of PNA probes

The PNA-hybridization probes *HP1*, *HP2* and *HP2’* (see Table 1) were synthesized on a ChemMatrix Rink amide solid support (Biotage) by manual 9-fluorenylmethoxycarbonyl (Fmoc) solid-phase synthesis as described in detail in Westerlund 2015^2^. Fmoc-protected PNA monomers, amino acids and (2-(2-aminoethoxy)ethoxy)acetic acid (AEEA), and the macrocyclic chelator 1,4,7,10-tetraazacyclododecane-1,4,7,10-tetraacetic acid (DOTA), were coupled in five times molar excess with five equivalents of the activator benzotriazole-1-yl-oxy-tris-pyrrolidino-phosphonium hexafluorophosphate (PyBOP) and five equivalents of *N, N*-diisopropylethylamine (DIEA) (0.2 M) in *N, N*-dimethylformamide (DMF). The coupling reactions were between 45 min and 2 h and were repeated if complete acylation of the amino groups had not been reached. Trifluoroacetic acid (TFA) cleavage of a small sample of resin followed by Matrix-Assisted Laser Desorption Ionization-Mass Spectrometry (MALDI-MS) (MALDI TOF/TOF analyzer, Sciex) analysis was performed to monitor the synthesis. Any remaining unreacted amino groups were capped with acetic anhydride for 10 min and the Fmoc groups were deprotected with 20% piperidine in N-methyl-2-pyrrolidone (NMP) for 60 min. The PNA probes were cleaved from the solid support with 95:2.5:2.5 of TFA/triisopropyl silane (TIS)/H_2_O for 3 h and extracted three times with diethyl ether. The secondary probe *HP2* was purified by reversed phase-high performance liquid chromatography (RP-HPLC). The *HP1* and *HP2’* probes were used for Sortase A-mediated conjugation to the Z domain without prior purification.

**Table 1:** PNA hybridization probes.

Name	Sequence	Theoretical MW (g mol^−1^)	Experimental MW (g mol^−1^)
*HP1*	G-G-G-S–S-a-g-t-c-t-g-g-a-t-g-t-a-g-t-c-E-K(DOTA)-AEEA-E-NH_2_	5398	5384
*HP2* *HP2’*	DOTA-AEEA-S–S-g-a-c-t-a-c-a-t-c–c-a-g-a-c-t-E-E-Y-NH_2_ G-G-G-AEEA-K(DOTA)-S–S-g-a-c-t-a-c-a-t-c–c-a-g-a-c-t-E-E-Y-NH_2_	5158 5072	5158 5055

### Expression, purification and labeling of Z_K35C-MBP_-SR-H_6_ and Z_35BPA_-SR-H_6_

Expression, purification and labeling of Z_K35C_-SR-H_6_ and Z_35BPA_-SR-H_6_ were done according to protocols described in detail earlier by our group^6,19^. Briefly, Z_K35C_-SR-H_6_ was expressed and immobilized metal affinity chromatography (IMAC)-purified using standard protocols for His_6_-tagged proteins. The lyophilized protein powder was resuspended in 50 mM phosphate buffer, 3 M guanidine hydrochloride, pH 8 with 20 mM dithiothreitol (DTT), and the protein was left to reduce for 1 h at 37 C. The buffer was then changed to phosphate-buffered saline (PBS) pH 6.6 using PD-10 desalting columns and 20-fold molar excess of 4-(*N*-maleimido)benzophenone (MBP; Sigma-Aldrich) was immediately added to the reduced protein. Following an overnight incubation at room temperature, excess MBP was removed using PD-10 columns equilibrated with 1 × sortase ligation buffer. Z_35BPA_-SR-H_6_ was expressed in 100 ml tryptic soy broth with yeast extract (TSBYE) cultures of Escherichia coli BL21 (DE3)* cells cotransformed with the pEVOL-pBpF plasmid, a gift from Peter Schultz, provided by Addgene (Addgene plasmid # 31190)^20^. Upon reaching an OD_600_ of 1, 0.5 mM of the unnatural amino acid, 4-benzoylphenylalanine (BPA, dissolved as 1 M in 1 M NaOH), was added to the culture, and protein expression was triggered by addition of 1 mM isopropyl β-d-1-thiogalactopyranoside (IPTG) and 0.2% arabinose. 4 h after induction another dose of BPA was added to the culture (final concentration 1 mM), and after another 22 h the culture was harvested by centrifugation. IMAC purification of Z_35BPA_-SR-H_6_ followed standard protocols for His-tagged proteins, and after purification the buffer was changed into 1 × sortase reaction buffer using PD-10 columns.

### Sortase A conjugation of Z domain to the PNA hybridization probes

Lyophilized PNA (*HP1* or *HP2’*) was dissolved in 10% dimethyl sulfoxide (DMSO)-H_2_O to a concentration of 1.6 mM and the pH was set to 7.5 with NaOH. 250 µl PNA (1.6 mM) and 250 µl Zx-SR-H_6_ (4 mM) (where Z_x_ represents Z_K35C-MBP_ or Z_35BPA_) were mixed with 45 µl 10 × sortase ligation buffer (0.5 M Tris–HCl, 1.5 M NaCl, 100 mM CaCl_2_), 10 µl Sortase A^3*^ (1 mM) and 20 µl nickel(II) acetate (100 mM). The volume was adjusted to 2 ml with 1 × sortase ligation buffer. The reaction mixture was incubated for 30 min at 37 °C on a rotating tube shaker and the reaction was stopped by the addition of 40 µl 10% TFA-H_2_O. The reaction mixture was added to an IMAC column with Talon metal affinity resin equilibrated with 1 × sortase ligation buffer. The product eluted with the flow-through, and was collected and purified by RP-HPLC (Agilent 1200 series, Agilent Technologies). The column used was a Zorbax 300SB-C18, 5 µm particle size, 9.4 × 250 mm, and the gradient was 0–58% acetonitrile in H_2_O with 0.1% TFA over 21 min at 70 °C for Z-*HP2’* and 0–65% acetonitrile in H_2_O with 0.1% TFA over 23 min at 70 °C for Z-*HP1*.

### Lactosamination of cetuximab

The monoclonal antibody cetuximab (Erbitux, Merck) was mixed with α-lactose-monohydrate (Sigma) and sodium cyanoborohydride (Aldrich) in 50 mM sodium tetraborate buffer (pH 9.2) to a final concentration of 32 µM, 28.4 mM and 148.2 mM respectively. The reaction was allowed to proceed for 4 h with shaking at 37 °C. The antibody solution was then purified using an Amicon Ultra-15 centrifugation filter with 50 kDa cut-off (Merck Millipore) and washed 5 times with 20 mM citrate–phosphate buffer, 150 mM NaCl, pH 6. To estimate the degree of modification, lactosaminated antibody and unmodified antibody were reduced by incubation with 50 mM DTT at 37 °C for 30 min followed by MALDI-MS (MALDI TOF/TOF analyzer, Sciex) analysis of the heavy and light chains.

### Site-specific photoconjugation of antibody to Z-PNA

The monoclonal antibody trastuzumab (Herceptin, Roche) was photoconjugated to Z*HP1* and lactosaminated cetuximab was photoconjugated to Z*HP2’* according to the method described in Westerlund et al., 2019^6^. For control experiments, non-lactosaminated cetuximab was photoconjugated to Z*HP1*, and lactosaminated cetuximab was photoconjugated to Z lacking the PNA probe, using the same protocol. In brief, 14.2 nmol antibody was mixed with 71 nmol Z-PNA and the volume was adjusted to 10 mL with 20 mM citrate–phosphate buffer, 150 mM NaCl, pH 6. The mixture was transferred to a sterile polystyrene dish and placed on ice. The photoconjugation was performed for 120 min under the illumination from UV-A lights. The pH was lowered after the reaction was finished by the addition of 10 mL 100 nM glycine–HCl, pH 3. The solution was centrifuged through an Amicon Ultra-15 centrifugation filter with 50 kDa cut-off and washed 5 times with glycine–HCl, pH 3. The pH was adjusted to 6 by the addition of storage buffer. The conjugation yield and final concentrations of lactosaminated cetuximab-Z*HP2’* (hereafter referred to as “CA”) and trastuzumab-Z*HP1* (hereafter referred to as “T-Z*HP1*”) were determined by SDS-PAGE analysis according to a previously developed method^6^ (see Supplementary Information).

### Analysis of the binding interaction between CA and T-Z*HP1*

Sodium dodecyl sulfate–polyacrylamide gel electrophoresis (SDS-PAGE) was used to assess the binding interaction between CA and T-Z*HP1* under reducing conditions. Trastuzumab, T-Z*HP1*, cetuximab, CA, and a sample of T-Z*HP1* pre-incubated with CA were applied to an NuPAGE 4–12% Bis–Tris gels (Thermo Fisher Scientific) and run at 200 V for 40 min at 4 °C under reducing conditions. The gel was stained using GelCode Blue Stain (Thermo Fisher Scientific) according to the manufacturer's instructions and scanned digitally.

### Radiolabeling chemistry

The primary targeting agent T-Z*HP1* was radiolabeled with [^131^I]NaI using direct radioiodination as described earlier^6^. In brief, 28 µg of T-Z*HP1* (0.167 nmol) in PBS was mixed with 4 µL (10 MBq) [^131^I]NaI. The reaction was initiated by adding chloramine-T (10 μL, 32 mg/mL in water). The mixture was carefully vortexed and incubated for 2 min at room temperature. Thereafter, the reaction was quenched by adding sodium metabisulfite (10 μL, 64 mg/mL in water) and the mixture was vortexed carefully. To be able to follow the biodistribution of the CA and the controls (cetuximab, cetuximab-Z*HP1* and cetuximab-lactose), the agents were radiolabeled with the radioisotope [^125^I]I by mixing 21 μg (0.12–0.14 nmol) of the conjugate in PBS with 9 μL [^125^I]NaI (10 MBq). Chloramine-T and sodium metabisulfite were added to the reaction mixture in the same order as described for T-Z*HP1*.

After the quenching of the reaction, a small aliquot from each reaction mixture (1 μL) was analyzed by radio-iTLC, eluted with 80% acetone. For purification a NAP-5 size exclusion column (GE Healthcare) pre-equilibrated with PBS was used.

### Evaluation of the interaction between [^131^I]I-radiolabeled T-Z*HP1* and [^125^I]I-radiolabeled CA using HER2-expressing SKOV3 cells

To demonstrate that the PNA-mediated interaction between CA and T-Z*HP1* was preserved after radiolabeling, four groups of HER2-expressing SKOV-3 cells (ca. 1 × 10^6^ cells/dish, 3 dishes per group) were used. Two groups were incubated with 10 nM of [^131^I]I-T-Z*HP1* at 4 °C for 1 h. Then, the medium containing [^131^I]I-T-Z*HP1* was removed and the cells were washed with serum-free medium. To confirm that the cell-associated radioactivity was a result of hybridization between the PNA probes in T-Z*HP1* and CA, dishes from one group were pretreated with 100 nM of *HP2*, non-labeled complementary PNA, after incubation with [^131^I]I-T-Z*HP1*. Thereafter, [^125^I]I-CA (10 nM) was added to both groups of cells and further incubated at 4 °C for 1 h. A third set of dishes contained only [^125^I]I-CA (no pre-incubation with [^131^I]I-T-Z*HP1*). To account for non-specific binding of [^125^I]I-CA, a fourth set of dishes was pre-incubated with a 100-fold excess of both trastuzumab (1 µM) and cetuximab (1 µM) prior to the addition of [^131^I]I-T-Z*HP1* and [^125^I]I-CA. After incubation with [^125^I]I-CA, media was collected, cells were detached by trypsin and the cell-associated radioactivity was measured using automated gamma-spectrometer.

### Animal studies

The animal experiments were planned and performed in accordance with national legislation on protection of laboratory animals. The animal studies were approved by the Local Ethics Committee for Animal Research in Uppsala (Uppsala djurförsöksetiska nämnd).

The dose (30 μg/0.17 nmol) of the CA was selected to be in excess with respect to circulating primary agent T-Z*HP1* (3 μg, 0.019 nmol).

To evaluate the influence of lactosamination and conjugation to PNA-modified Z-domain on cetuximab biodistribution, sixteen female NMRI mice (average weight 26 ± 2 g) were randomized to four groups with four mice each. The first group (n = 4) of mice was intravenously injected with 20 kBq [^125^I]I-cetuximab, 30 μg in 100 μL 2% bovine serum albumin (BSA) in PBS. Mice from two other groups were injected with either the lactosaminated mAb, [^125^I]I-cetuximab-lactose, or the Z domain-modified mAb, [^125^I]I-cetuximab-Z*HP1* (20 kBq, 30 μg in 100 μL 2% BSA in PBS). The last group of mice was injected with 20 kBq [^125^I]I-CA containing both lactosaminated residues and PNA-modified Z domain (30 μg in 100 μL 2% BSA in PBS). Mice were anesthetized by an intraperitoneal injection of ketamine and xylazine solution, sacrificed and euthanized by cervical dislocation 1 h post-injection of agents. Blood, liver and other organ and tissue samples were collected, weighed and measured for radioactivity using an automated gamma-spectrometer along with standards to determine the percentage of injected dose per gram (%ID/g).

To evaluate T-Z*HP1* blood clearance with and without CA administration, eight NMRI mice were divided into two groups (n = 4). Both groups were injected with [^131^I]I-labeled T-Z*HP1* (20 kBq, 3 μg/0.017 nmol in 100 μL 2% BSA in PBS per mouse). Six hours after T-Z*HP1* injection, one group of mice received an intravenous injection of [^125^I]I-CA (20 kBq, 30 μg/0.17 nmol in 100 μL 2% BSA in PBS per mouse). The other group was injected intravenously with PBS solution only. Thereafter, mice were euthanized by cervical dislocation 1 h post CA or PBS injection, and organs were collected and measured for radioactivity as mentioned above.

Blood samples from mice in the two biodistribution studies were collected and submitted to centrifugation at 10,000 rpm for 10 min at 4 °C to separate their components. The obtained serum samples (ca. 0.5 mL) were then applied to a NAP-5 size exclusion column (GE Healthcare) pre-equilibrated with 2% BSA in PBS. Columns were eluted with 2% BSA in PBS into two fractions of radioactive components: high molecular weight (HMW) compounds fraction (> 5 kDa), and low molecular weight (LMW) compounds fraction (< 5 kDa). The radioactivity was measured using an automated gamma-spectrometer.

## Results

### Synthesis and purification of PNA probes

The PNA probes *HP1* and the complementary *HP2* and *HP2’* (see Table 1) were successfully synthesized manually with Fmoc chemistry. The intermediate products were analyzed by mass spectrometry after the coupling of each amino acid or PNA monomer. For several residues the coupling step was repeated as the reaction had not run to completion. Any remaining amino groups after repeated coupling were capped with acetic anhydride to avoid the formation of deletion peptides. After TFA cleavage of the probes, *HP2* was purified by HPLC to a final purity of > 95%. The *HP1* and *HP2’* probes could be used in the Sortase A conjugation reaction without removing the terminated side products by HPLC purification, as only the full-length probes carrying N-terminal glycine residues are substrates for the enzyme.

### Sortase A conjugation of Z domain to the PNA hybridization probes

The Sortase A-mediated reaction was performed in sortase A ligation buffer and was stopped by lowering the pH by addition of acid. After conjugation, an IMAC purification step was performed to remove the His-tagged enzyme and any unreacted Z_x_-SR-H_6_ protein. Unreacted PNA and hydrolyzed side product, both co-eluting with the Z-PNA conjugate, could conveniently be separated from the desired Z-PNA product by HPLC purification (see Fig. S1 for a representative HPLC chromatogram). MALDI-MS was used to confirm the correct Z-PNA conjugates after purification (see Table 2 and Figs. S2A-C). The Z-PNA products produced by conjugation of Z_K35C-MBP_-SR-H_6_ and Z_35BPA_-SR-H_6_, where the photoactivatable benzophenone group was introduced by maleimide coupling to the side chain cysteine or by direct incorporation of benzoylphenylalanine using an Amber suppression system, respectively, were shown to have the same antibody photoconjugation efficiencies (see Fig. S5B-C) and were used interchangeably in this study.

**Table 2:** Proteins and protein-PNA conjugates.

Name	Theoretical MW (g mol^−1^)	Experimental MW (g mol^−1^)
Z_K35C-MBP_-SR-H_6_	8710	8726
Z_35BPA_-SR-H_6_	8788	8765
Z_K35C-MBP_-*HP1*	13,412 (13,430)^a^	13,434
Z_K35C-MBP_-*HP2*’	13,086 (13,104)^a^	13,073
Z_35BPA_-*HP2’*	12,906	12,881

^a^Theoretical MW after hydrolysis of the maleimide ring^31^.

### Lactosamination of cetuximab

Lactosamination was performed by reductive amination of the lysine groups on cetuximab to create a covalent bond between the lactose moiety and the primary amino groups on lysine side chains and polypeptide N-termini (see Fig. S3). The lactosamination protocol was based on a published method^17^ and was optimized for this application by varying the concentration of reagents, temperature and reaction time to give a suitable degree of antibody modification.

To determine the degree of modification, the antibody was reduced by DTT and analyzed by MALDI-MS. The MALDI spectra of the antibody heavy chains (HC) (see Fig. S4A) yielded single, broad peaks both before and after lactosamination, and the resolution was too poor to separate the peaks corresponding to different number of lactose units. The number of lactose modifications was therefore calculated by dividing the average mass difference between the modified and unmodified heavy chain by the mass of a conjugated lactose unit. The MALDI spectra of the antibody light chains (see Fig. S4B) showed separation between the peaks corresponding to different numbers of lactose units. The heights of the peaks were assumed to correspond to the relative abundance of the different molecules. The number of lactose modifications for the light chains was calculated from the relative abundance of the different lactosaminated species.

Using the optimized protocol, the antibody lactosamination was performed for 4 h at 37 °C and gave an average of 35 lactose units conjugated to cetuximab.

### Site-specific cross-linking of Z-PNA to cetuximab and trastuzumab

The antibody was photoconjugated to Z-PNA according to an earlier described protocol^6^. After photoconjugation the pH was lowered to 3, which was shown to increase the solubility of the antibody-PNA complex. It has previously been shown that the trastuzumab antibody is compatible with acidic conditions, such as acidic elution in affinity chromatography purification, using 0.1 M glycine–HCl (pH 2.5–3.3) or 0.1 M citric acid (pH 2.5–3.5) buffers^21^. We have also shown by SPR that PNA-conjugated trastuzumab retains sub-nanomolar affinity to HER2 after purification at pH 3^6^. The photoconjugation yield was determined by SDS-PAGE analysis and calculated from the relative intensities of modified and unmodified heavy chains (see Fig. S5A-C). The conjugation yield was approximately 45% (ranging from 43 to 46% for different conjugation reactions) for both CA and T-Z*HP1*.

### SDS-PAGE analysis of the binding interaction between CA and T-Z*HP1*

**Figure 3:**
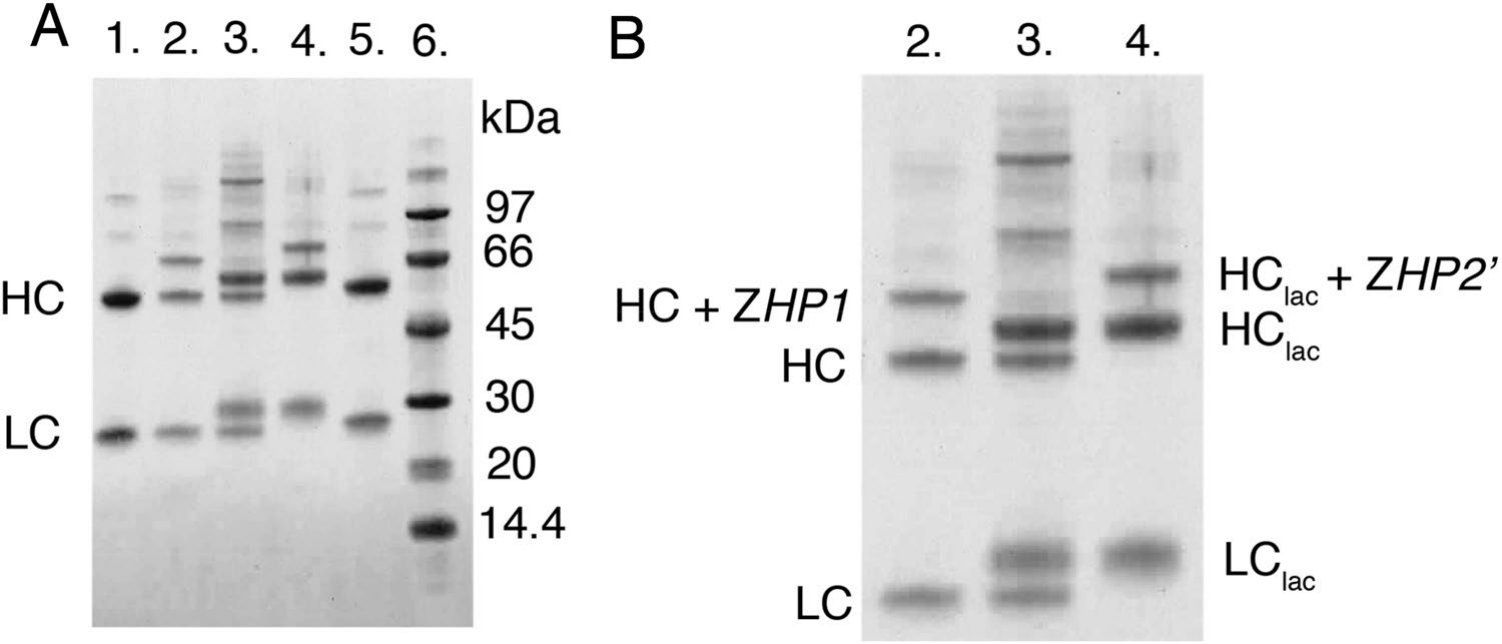
SDS-PAGE analysis of the binding between CA and T-Z*HP1*. **(A)** Lane 1: trastuzumab, lane 2: T-Z*HP1*, lane 3: T-Z*HP1* pre-incubated with CA, lane 4: CA, and lane 5: cetuximab. **(B)** Close up of lanes 2–4. The gels shown have been cropped for clarity; the original SDS-PAGE gel is shown in Supplementary Fig. S7.

Preincubation of CA with T-Z*HP1* resulted in the disappearance of bands corresponding to antibody HC subunits conjugated with Z-PNA, and the appearance of new protein bands with higher apparent molecular weights (Fig. 3, lane 3). In contrast, bands corresponding to antibody light chains and unmodified heavy chains remained unshifted.

### Radiolabeling chemistry

Radioiodination of both conjugates T-Z*HP1* and CA was successful. The radiochemical yields were 92 ± 5% and 90 ± 7% for T-Z*HP1* and CA, respectively. The radiochemical yield for agents used as control were 92 ± 1% for cetuximab, 93 ± 0% for cetuximab-Z*HP1* and 75 ± 12% for cetuximab-lactose. Purification using NAP-5 size exclusion chromatography provided a radiochemical purity of more than 95% for all studied constructs before continuing with animal studies.

**Figure 4:**
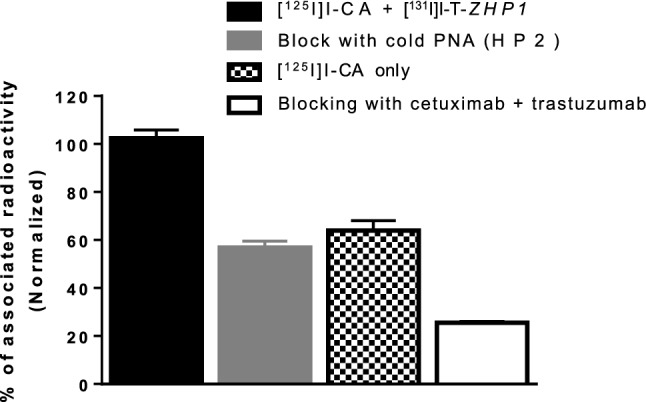
In vitro reactivity of [^125^I]I-CA with [^131^I]I-T-Z*HP1* bound to HER2-expressing SKOV-3 cells. Cells were first incubated with [^131^I]I-T-Z*HP1* at 4 °C for 1 h. Thereafter an equivalent amount of the [^125^I]I-CA was added and cells were further incubated at 4 °C for 1 h. Media and cells were collected and measured for radioactivity. To determine the specificity of the *HP1*-*HP2* hybridization, cells in group 2 (grey) were incubated with an excess of unlabeled *HP2* after incubation with [^131^I]I-T-Z*HP1* and prior to the addition of [^125^I]I-CA. [^125^I]I-CA was directly added to cells in group 3 (semi filled) without preincubation with [^131^I]I-T-Z*HP1*. To account for the [^125^I]I-CA specific binding to the SKOV-3 cells (also expressing EGFR), the cells in group 4 (empty) were incubated with 100-fold molar excess of cetuximab in order to block the EGFR-receptors prior to the addition of the [^125^I]I-CA.

To check if the reactivity of [^125^I]I-CA to [^131^I]I-T-Z*HP1* was preserved, the binding specificity assay using HER-2 expressing SKOV3 cells was used (Fig. 4). The binding of [^125^I]I-CA to cell-bound [^131^I]I-T-Z*HP1* exceeded that in other control groups by ca. 1.5–4-fold (p < 0.05). This binding was significantly reduced when an excess of the complementary PNA, *HP2*, was added to [^131^I]I-T-Z*HP1* before the addition of [^125^I]I-CA. Binding was also reduced when no [^131^I]I-T-Z*HP1* was added or when binding of [^131^I]I-T-Z*HP1* and [^125^I]I-CA was prevented by saturation of HER2 and EGFR receptors using non-labeled parental mAbs, trastuzumab and cetuximab.

### Animal studies

**Figure 5:**
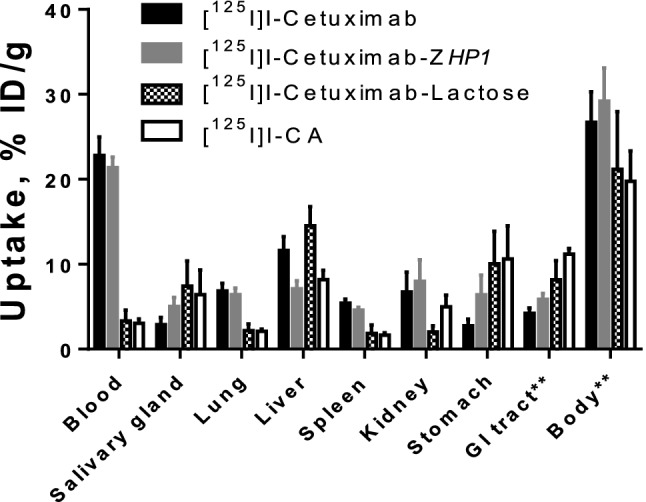
Biodistribution and blood clearance of [^125^I]I-labelled cetuximab, cetuximab-Z*HP1,* lactosaminated cetuximab (cetuximab-lactose) and CA in NMRI normal mice. Mice were sacrificed 1 h post injection of the respective mAbs (33 µg/mouse). Data is presented as an average value from four animals ± standard deviation. ** Uptake in gastrointestinal tract (GI tract) and remaining body is presented as %ID per whole sample.

The results of the comparative biodistribution of [^125^I]I-labelled cetuximab, cetuximab-lactose, cetuximab-Z*HP1* and the CA in normal female NMRI mice is summarized in Fig. 5.

The data obtained shows a seven to eightfold decrease in the blood level of radioactivity for the lactosaminated mAbs (cetuximab-lactose and the CA) in comparison with the parental antibody cetuximab or cetuximab-*ZHP1* as early as 1 h p.i. Lactosamination of cetuximab in cetuximab-lactose also significantly increased the liver uptake compared to non-lactosaminated native antibody cetuximab. This effect was not observed for the CA, i.e. containing both lactose and PNA-modified Z-domain. Radioactivity accumulation in the lungs and spleen was also significantly lower for the lactosaminated variants.

To confirm that the rapid clearance of CA is mainly due to lactosamination of cetuximab, the biodistribution of the non-lactosaminated, PNA-modified Z domain-containing control, cetuximab-Z*HP1,* was also evaluated. The data obtained shows no significant difference in blood and the majority of the normal organs radioactivity uptake levels between cetuximab-Z*HP1* and cetuximab (Fig. 5). The only exceptions were liver and stomach.

**Figure 6:**
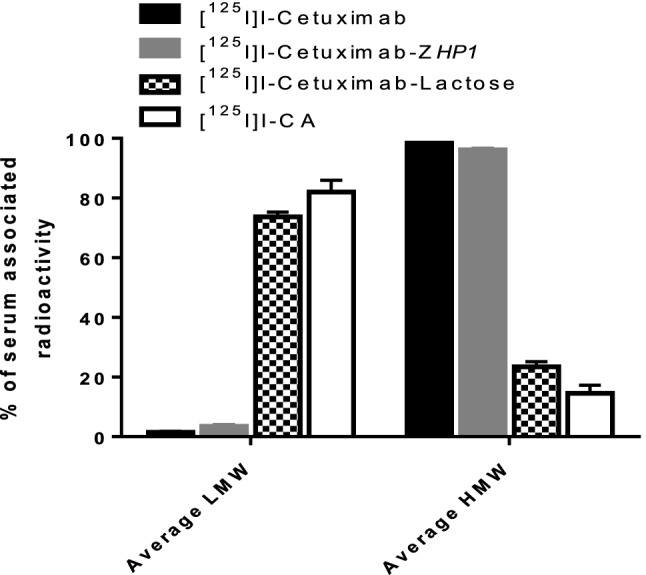
Analysis of NMRI normal mice blood serum using NAP-5 size exclusion chromatography 1 h after mice were injected with [^125^I]I-labelled cetuximab, cetuximab-Z*HP1,* cetuximab-lactose and CA. Data are presented as % of total serum associated radioactivity (average of three samples). LMW represents radiocatabolites with the molecular weight of less than 5 kDa. HMW represents intact antibodies.

Serum analysis using NAP-5 size exclusion chromatography (Fig. 6) revealed that the majority of blood-borne radioactivity (82 ± 4% of total serum-associated radioactivity) 1 h after injection of [^125^I]I-CA was present in the form of radiocatabolites with the molecular weight of less than 5 kDa i.e. eluted in the LMW fraction. This was also the case for cetuximab-lactose. On the opposite, the majority of the blood-borne radioactivity (98 ± 1.5% of total serum-associated radioactivity) following [^125^I]I-cetuximab injection was present in the form of intact antibodies eluted in the HMW fraction.

**Figure 7:**
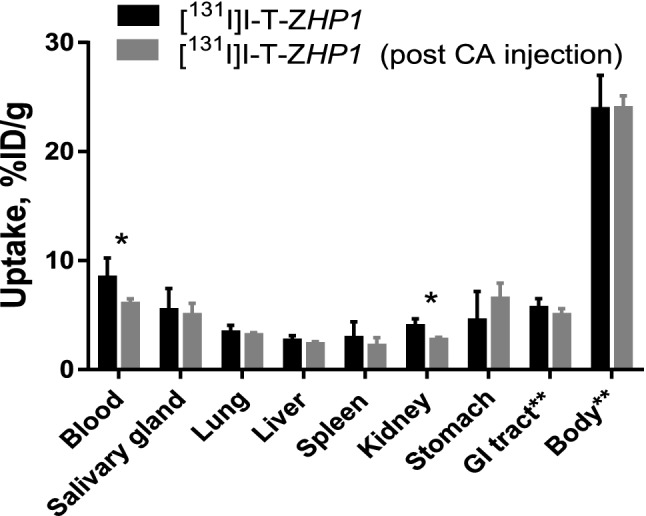
Blood and body clearance of [^131^I]I-T-Z*HP1* with and without use of CA. NMRI normal mice were injected with 3 μg of T-Z*HP1* per mouse followed by 30 μg of the CA 6 h later. Mice were sacrificed 1 h post CA injection. Data is presented as an average value from four animals ± standard deviation. Asterisks (*) indicate significant difference (p < 0.05 in paired Student´s t-test). ** Uptake in gastrointestinal tract (GI tract) and remaining body is presented as %ID per whole sample.

**Figure 8:**
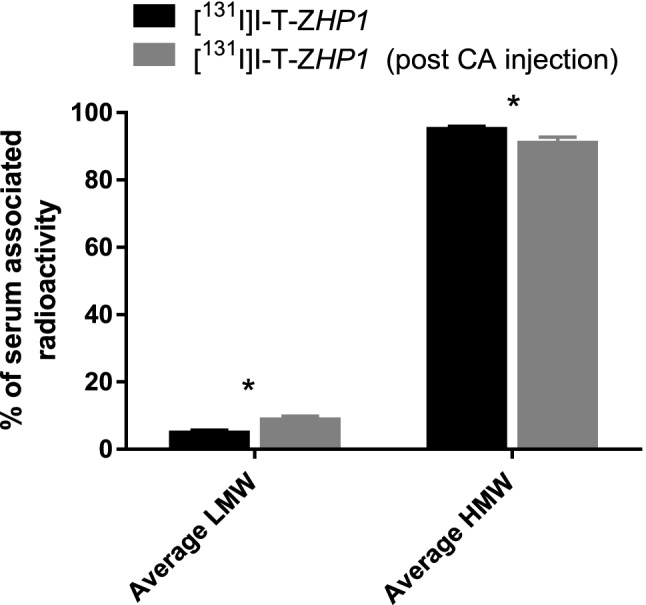
Analysis of NMRI normal mice blood serum using NAP-5 size exclusion chromatography when mice were injected with [^131^I]I-T-Z*HP1* only (black) or [^131^I]I-T-Z*HP1* followed by the injection of CA 6 h later (grey). Data are presented as % of total serum associated radioactivity (average of three samples). LMW represents radiocatabolites with the molecular weight of less than 5 kDa.

To test whether the CA would effectively remove residual amounts of the circulating primary targeting agent, 3 μg of [^131^I]I-T-Z*HP1* was i.v. injected to NMRI mice followed by injection of [^125^I]I-CA (30 μg) 6 h later. Results from the biodistribution study (1 h post-CA injection) demonstrated that [^131^I]I-T-Z*HP1*, without CA, exhibited a high blood radioactivity level as expected (8.5 ± 1.8%ID/g; Fig. 7). One hour post [^125^I]I-CA injection, the [^131^I]I-T-Z*HP1* blood levels was significantly (p < 0.05) reduced, 1.4-fold (8.5 ± 1.8 to 6 ± 0.4%ID/g, Fig. 7). The study also showed minor radioactivity accumulation in the liver and spleen post-[^131^I]I-T-Z*HP1* injection with and without injection of the CA (2.7 ± 0.4 and 2.3 ± 0.2%ID/g in the liver, respectively). The biodistribution of [^125^I]I-CA injected 6 h post-[^131^I]I-T-Z*HP1* administration was similar to that observed for the CA alone in the previous experiment (Figs. 5 and 6). Analysis of blood-borne radioactivity demonstrated that 90.8 ± 1.8% of the total serum associated radioactivity post-[^131^I]I-T-Z*HP1* followed by injection of the CA, was presented in the high molecular weight form (Fig. 8). The percentage of high-molecular weight form was significantly higher (p < 0.05) in the control group, where no CA was injected after [^131^I]I-T-Z*HP1* (94.8 ± 1.8% of total serum associated radioactivity).

## Discussion

Pretargeting is a promising approach to reduce the background resulting from slow blood clearance of intact antibodies in immunoPET and radioimmunotherapy. The concept is based on the separation of the tumor-targeting step from the administration of the radiolabeled probe, and relies on in vivo association of the primary and secondary agents, by high-affinity interactions such as the interaction between biotin/(strept)avidin, bispecific antibodies binding to radiolabeled haptens, and hybridization of complementary oligonucleotides, or bioorthogonal chemical reactions, such as tetrazine ligation^22^. To further enhance the contrast between tumor and non-tumor organs, a clearing agent, or a “chase” molecule, can be included in the pretargeting system for removal of circulating primary agent from the blood. The clearing agent is typically based on a bulky protein, which is functionalized for specific capture of the primary pretargeting agent. Examples include human transferrin functionalized with a metal chelate^23^ and galactose-modified albumin conjugated to tetrazine^10^. Alternatively, the clearing agent can be based on a polymer, such as dextran^24^ or, as recently described, a synthetic glycodendrimer displaying terminal α-thio-*N*-acetylgalactosamine moieties^25^. A prerequisite for an efficient clearing agent is that it should rapidly bind the primary targeting agent in blood and remove it from circulation without saturating the tumor. The removed complex should be directed towards the liver in a specific manner to enhance hepatic catabolism and reduce unnecessary accumulation of the removed complex in non-targeted normal tissues. Moreover, the clearing agent should be bulky enough to have reduced extravasation to the tumor. The CA described here fits well into these criteria having molecular weight over 170 kDa, rapid elimination from blood, and specific uptake in liver.

The CA developed in this study was produced by lactosamination of a therapeutic monoclonal antibody followed by site-specific conjugation to a PNA probe, which is complementary to the primary agent. Cetuximab is an EGFR-targeting chimeric mouse/human antibody approved for treatment of colorectal cancer, non-small cell lung cancer, and head and neck cancer. It was chosen as the starting material for development of the CA mainly because it is a bulky protein with a favorable safety profile, already approved for use in humans. An additional advantage of cetuximab is that the conjugation of PNA probes to the antibody could be performed using the same photoconjugation protocol employed for preparation of the primary targeting agent, thereby streamlining the production of the different components of the pretargeting system. The site-specific and covalent conjugation of oligonucleotide probes to antibodies using an Fc-binding Z domain functionalized with a photoactivatable benzophenone group has earlier been explored in our group for preparation of antibody-DNA^19^ and antibody-PNA^6^ conjugates. The method relies on the presence of a benzophenone group in the Z domain, which upon irradiation with UV light is activated and can form a covalent bond with amino acids in close proximity, with a preference for methionine residues^26^. The benzophenone group can be introduced in the Z domain by chemoselective conjugation, e.g. by reaction of a maleimido-benzophenone reagent to the thiol side chain of cysteine, or by incorporation of the unnatural amino acid benzoylphenylalanine, e.g. by using an amber tRNA suppression system. Advantages of the method include that conjugation is directed to a well-defined binding site in the Fc part of the antibody, not interfering with the target recognition, and that high photoconjugation efficiencies are reproducibly obtained for labeling human IgG1 antibodies. In this study, PNA-labeling of the primary targeting agent T-Z*HP1* and the CA both resulted in approximately 45% modification of the heavy chains, which is close to on average one label per antibody as the IgG molecule is a symmetrical dimer.

Lactosamination of the antibody was performed to promote liver clearance of the CA by targeting the asialoglycoprotein receptors in hepatocytes^12^. The lactose moieties were conjugated by reductive amination to the primary amino groups of the lysine side chains and the N-termini of the heavy and light chains. Based on the crystal structure of the B domain (from which the Z domain is derived) in complex with the Fc region of IgG (PDB file 5u4y) it appears that the lysine residues in the Fc part of the antibody are not directly involved in the binding interaction (see Fig. S6), but it cannot be excluded that excessive lactosamination of the antibody would slightly interfere with the binding and/or photoconjugation of the Z domain. In order to reduce the risk of disrupting the antibody structure and function, the lactosamination protocol was optimized to give a moderate degree of modification. The optimized protocol gave an estimated number of 35 lactose residues, which corresponds to the modification of 38% of the available amino groups in cetuximab (88 lysines plus 4 N-terminal amino groups on the heavy and light chains)^27^.

The SDS-PAGE analysis of in vitro pre-reacted T-Z*HP1* and CA demonstrated that the Z-PNA-modified HC subunits of each protein were able to react with each other. The interaction was stable enough to withstand the conditions under which the reducing SDS-PAGE gel was run, and was probably mediated through *HP1*-*HP2’* PNA-PNA hybridization.

To study if the radiolabeling of [^131^I]I-T-Z*HP1* and [^125^I]I-CA had an impact on the reactivity between T-*ZHP1* and CA, a binding specificity experiment using HER2-expressing SKOV3 cells was performed. Results from the in vitro experiment clearly demonstrated that radiolabeled [^131^I]I-T-Z*HP1* and [^125^I]I-CA retained the capacity to react with each other in a PNA-PNA dependent manner (Fig. 4). Binding of [^125^I]I-CA to cells was significantly reduced when no primary agent T-Z*HP1* was added or when T-Z*HP1* was saturated with complementary PNA, *HP2*, prior to the addition of the CA.

Results from the biodistribution study in normal NMRI mice demonstrated that lactosaminated cetuximab (cetuximab-lactose) cleared from the blood to the liver more rapidly than the parental mAb cetuximab as early as 1 h p.i. The effect of lactosamination was retained even after functionalizing the cetuximab with PNA (CA) through site-specific coupling of PNA-modified Z-domain to the Fc-domain of cetuximab (Fig. 5). The concentration of radioactivity in the blood after injection of cetuximab-lactose and CA injection was 2.7 ± 0.1 and 3.0 ± 0.5%ID/g, respectively. This indicates that the Fc-domain modification had no impact on CA localization in the liver. The liver uptake of CA was however lower than the liver uptake of cetuximab-lactose and it can be speculated that this is due to interference of the binding of the Fc-region of the CA to the neonatal FcRn receptors in hepatocytes. It has previously been reported that FcRn is expressed in hepatic endothelium and hepatocytes^28^ and that blockade of FcRn should result in degradation of radiolabeled IgG and radiometal accumulation in the liver^29,30^. In the current study, the CA was labelled with the non-residualizing radiohalogen ^125^I, which generates leaky radiocatabolites that diffuse out of the liver and redistribute to other body organs. This is supported by results from Fig. 5, where there is a lower accumulation of radioactivity in liver post CA-injection, but significantly higher radioactivity concentration in the kidneys and GI-tract compared to [125I]I-cetuximab-lactose. In Fig. 6 it can be seen that there are significantly higher levels of low molecular weight (LMW) radiocatabolites in the blood-borne radioactivity for [125I]I-CA compared to [125I]I-cetuximab-lactose. Without the galactose units on the CA, cetuximab-Z-PNA had a cetuximab-like biodistribution with a high blood radioactivity of 21.4 ± 1.2% ID/g. It is important to mention that the chimeric mAb cetuximab is not cross-reactive to murine EGFR and hence the observed liver uptake is mainly asialoglycoprotein receptor-mediated. In humans, binding to hepatic EGFR may provide an additional mechanism to further enhance the plasma clearance of the complex. It is also important to note that lactosamination is a relatively mild chemical procedure, and hence the observed enhanced kinetics and elevated liver uptake cannot be attributed to denaturation of cetuximab and trapping by the reticulo-endothelial system, in particular the Kupffer cells of the liver. Analysis of the blood-borne radioactivity post CA injection revealed that most of the total radioactivity was present in the form of radiocatabolites with the molecular weight of less than 5 kDa (LMW fraction). This was not the case for cetuximab where over 80% of the total blood-borne radioactivity was present in the high molecular weight form (HMW fraction). These results clearly demonstrate the immediate processing of the CA by hepatocytes followed by hepatic catabolism and excretion of the catabolites to the blood circulation.

Injection of the CA 6 h post injection of the primary agent T-Z*HP1* resulted in a significant (p < 0.05) increase in clearance of T-Z*HP1* from the blood compared to T-Z*HP1* alone (Fig. 7), however, with a modest impact. The radioactivity concentration in the blood after [^131^I]I-T-*ZHP1* injection followed by CA was reduced by ca. 1.4-fold from 8.5 ± 1.8 to 6 ± 0.4%ID/g. A surprising finding was that the enhanced clearance of T-Z*HP1* (post CA injection) was not accompanied by an increase in the liver uptake (Fig. 7). One explanation could be that the complex is rather quickly processed by the hepatocytes, and that the catabolites are released to the circulation again in a very rapid manner, i.e. in less than 1 h. It is also known that the non-residualizing labels like [^131^I]I and [^125^I]I form leaky catabolites after lysosomal degradation that diffuse freely through the cell membrane. However, analysis of the radioactivity content of the blood using size exclusion chromatography revealed that this was not the case and that the majority of the blood-borne radioactivity post [^131^I]I-T-Z*HP1* injection followed by the CA, was present in the form with a molecular weight of more than 5 kDa, i.e. intact [^131^I]I-T-Z*HP1* (Fig. 8). On the other hand, there was a significant increase in the amount of LMW (< 5 kDa) radiocatabolites in the blood post-CA injection, suggesting that a fraction of [^131^I]I-T-Z*HP1* was directed to the liver by the action of the CA, where it was degraded, resulting in leakage of radiocatabolites to the blood, and partial redistribution to other organs. The impact of this effect was not as high as expected. We are aware that this moderate effect of the CA would likely not be sufficient to remove meaningful amounts of circulating T-Z*HP1* from the blood. However, the obtained results provide an initial proof-of-principle, which could guide future development of a more efficient CA.

A possible explanation of incomplete removal of T-Z*HP1* from the circulation using CA may stem from the fact that the in vitro studies of the binding between CA and T-Z*HP1* were performed under conditions that favor the reaction between two molecules. However, these conditions are greatly influenced by the high dilution encountered in vivo. The amount of injected CA in the current study was in ninefold molar excess to that of T-Z*HP1* and the gap between the injections was 6 h. It would therefore be logical to assume that any further increase in the injected amount of CA may not result in any enhanced efficacy, as the current settings would already provide meaningful proportions between CA and T-Z*HP1,* for the reaction to occur. A more efficient strategy could be to increase the number of complementary PNA molecules (*HP2*) per cetuximab molecule in the CA. This would probably enhance the chances of interaction between the CA and T-Z*HP1* and facilitate the removal of the residual primary agent prior to the injection of the effector *HP2*. Rossin and coworkers have followed a similar strategy when developing a clearing agent^10^. Despite the fast association rates between tetrazine and TCO (ranging from 5 × 10^5^ to 7.5 × 10^7^ M^−1^ s^−1^), the group has developed a clearing agent based on an albumin scaffold carrying 9–13 tetrazine moieties for reaction with the mAb-bound TCO. It was possible to reduce the mAb-TCO blood concentration by eightfold after a single injection of the clearing agent. We are now planning to pursue a similar strategy. One possible approach could be the direct coupling of PNA molecules to available amine residues in the mAb not occupied by the galactose residues.

In conclusion, this study provided a proof-of-principle for the molecular design of a CA for antibody-based PNA-mediated pretargeting. However, the effect was moderate and further studies are required to enhance this effect.

## Supplementary information

Supplementary Information.
