# Supplementary material for: Evaluation of an antibody-PNA conjugate as a clearing agent for antibody-based PNA-mediated radionuclide pretargeting
Source: Sci Rep. 2020 Nov 27;10:20777. doi: 10.1038/s41598-020-77523-y (PMC7695838; doi:10.1038/s41598-020-77523-y)
Supplement: Supplementary file 1 — Supplementary Information. [file 41598_2020_77523_MOESM1_ESM.docx]

**SUPPLEMENTARY INFORMATION**

**Evaluation of an antibody-PNA conjugate as a clearing agent for antibody-based** **PNA-mediated radionuclide pretargeting**

Anders Myrhammar**;** Anzhelika Vorobyeva; Kristina Westerlund; Shuichiro Yoneoka; Anna Orlova; Takehiko Tsukahara; Vladimir Tolmachev; Amelie Eriksson Karlström; Mohamed Altai

***Analysis of the products formed by Sortase A conjugation of Z domain to the PNA hybridization probes***


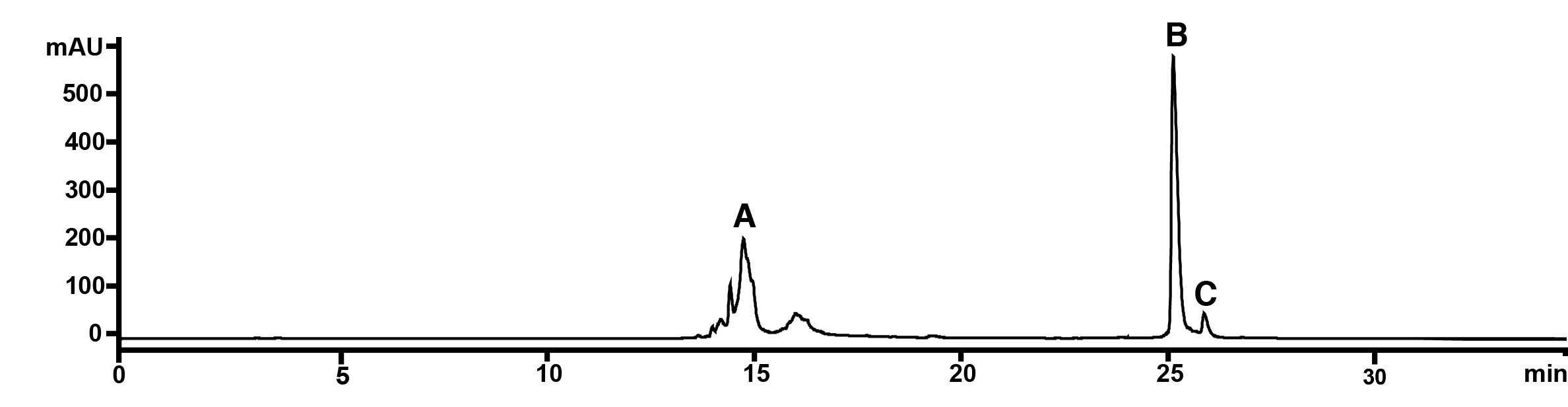


Fig. S1. HPLC purification of Z_35BPA_-*HP2’* after sortase A conjugation. Peak A is unpurified, unreacted PNA, peak B is the correct product Z_35BPA_-*HP2*’ and peak C is unreacted Z_35BPA_ (hydrolysis side product).


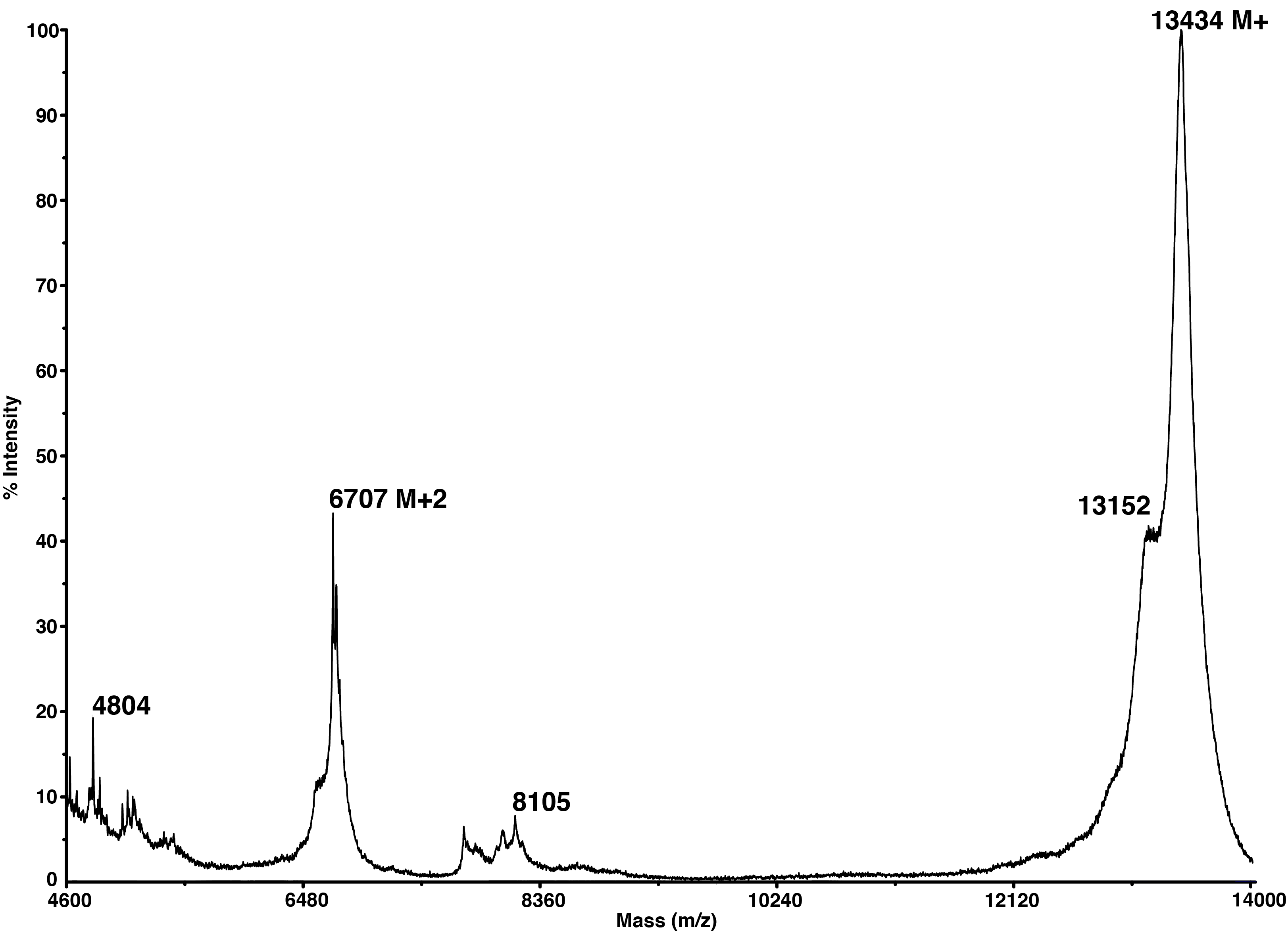


Fig. S2A) MALDI-MS spectrum of HPLC-purified Z_K35C-MBP_-*HP1*. Theoretical molecular weight 13 412 Da (or 13 428 Da after hydrolysis of the maleimide ring [1]); experimental molecular weight 13 434 Da.


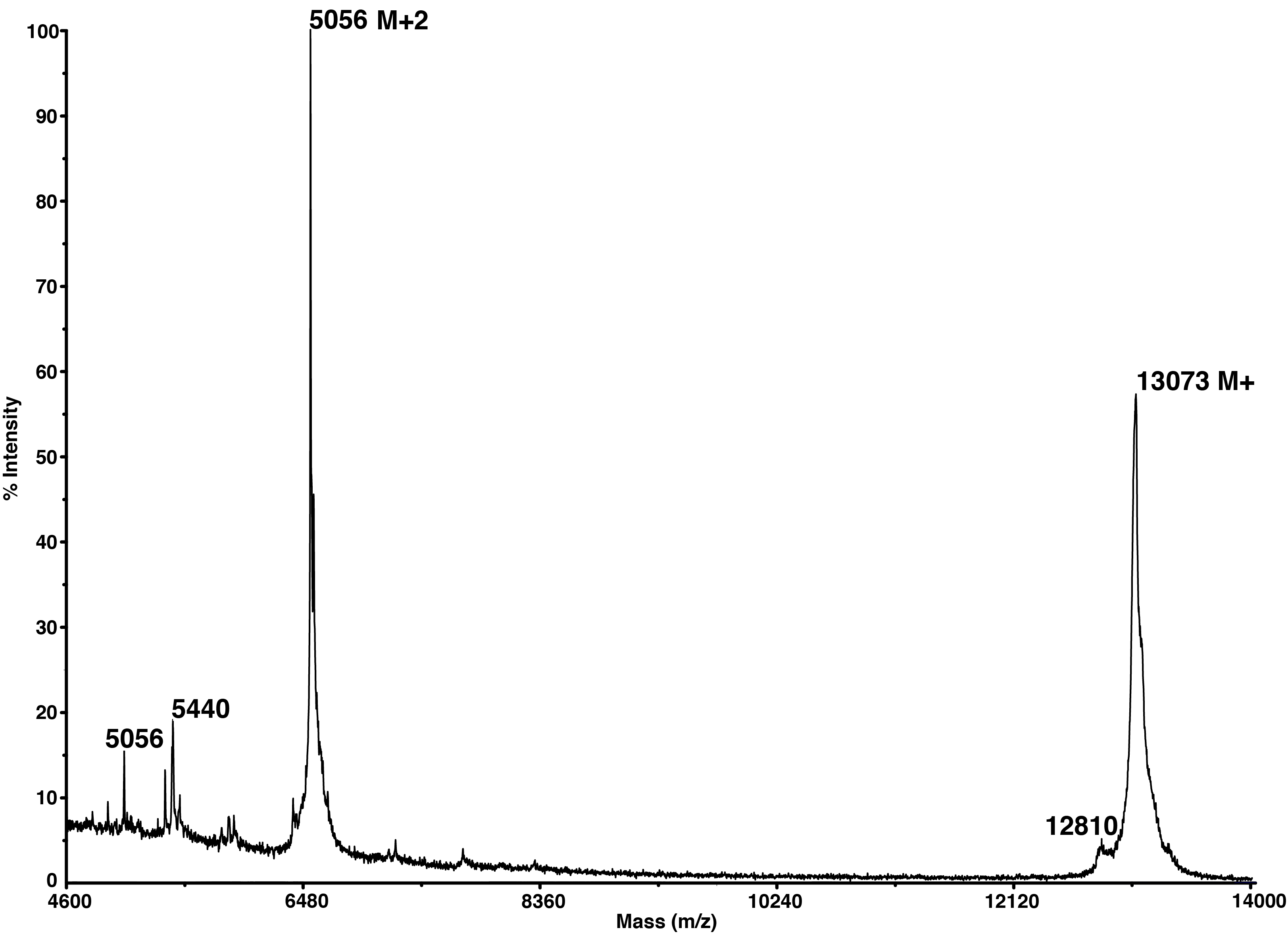


Fig. S2B) MALDI-MS spectrum of HPLC-purified Z_K35C-MBP_-*HP2’.* Theoretical molecular weight 13 086 Da (or 13 104 Da after hydrolysis of the maleimide ring [1]); experimental molecular weight 13 073 Da.


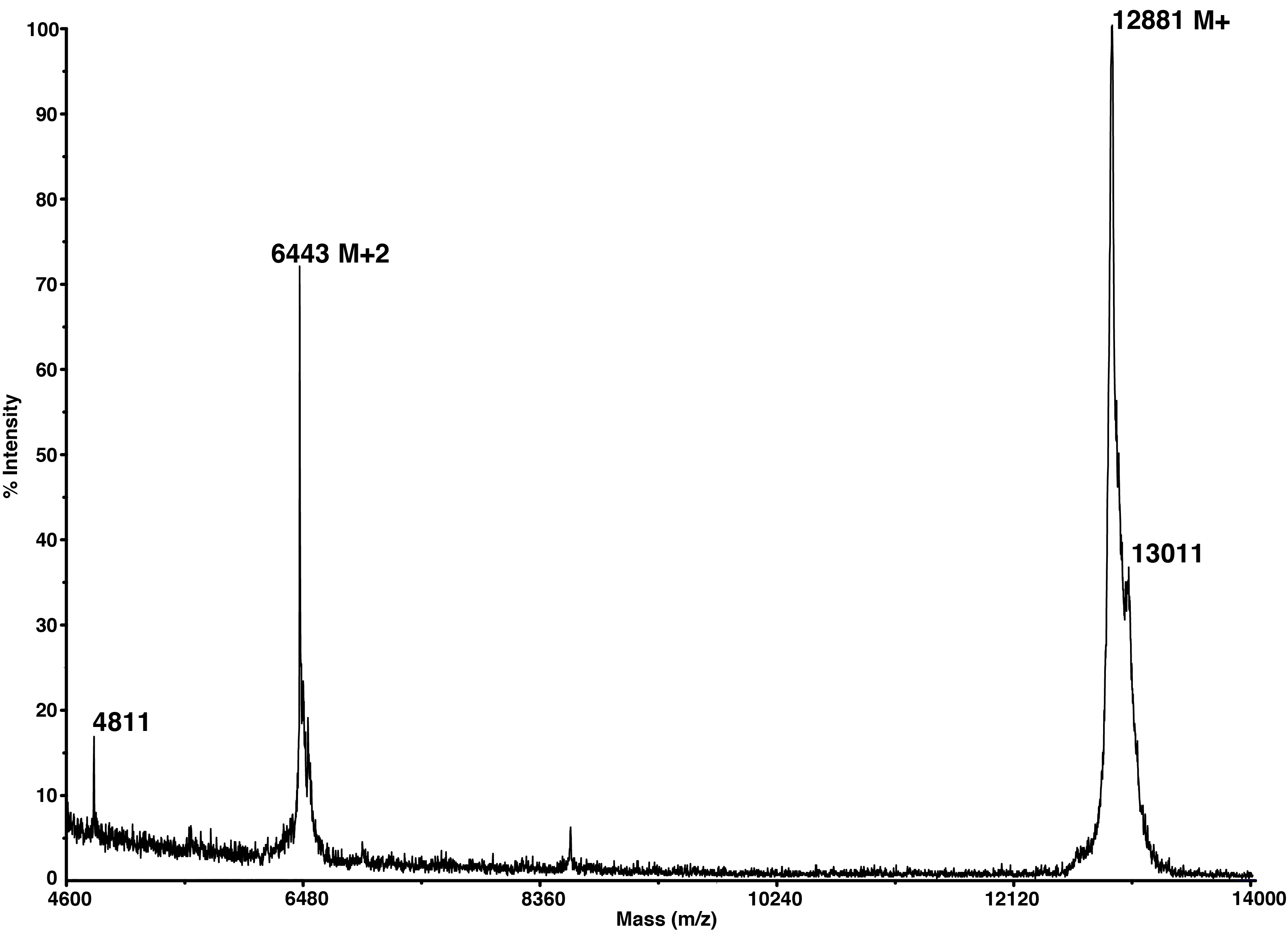


Fig. S2C) MALDI-MS spectrum of HPLC-purified Z_35BPA_-*HP2’.* Theoretical molecular weight 12 906 Da; experimental molecular weight 12 881 Da.

***Antibody lactosamination***


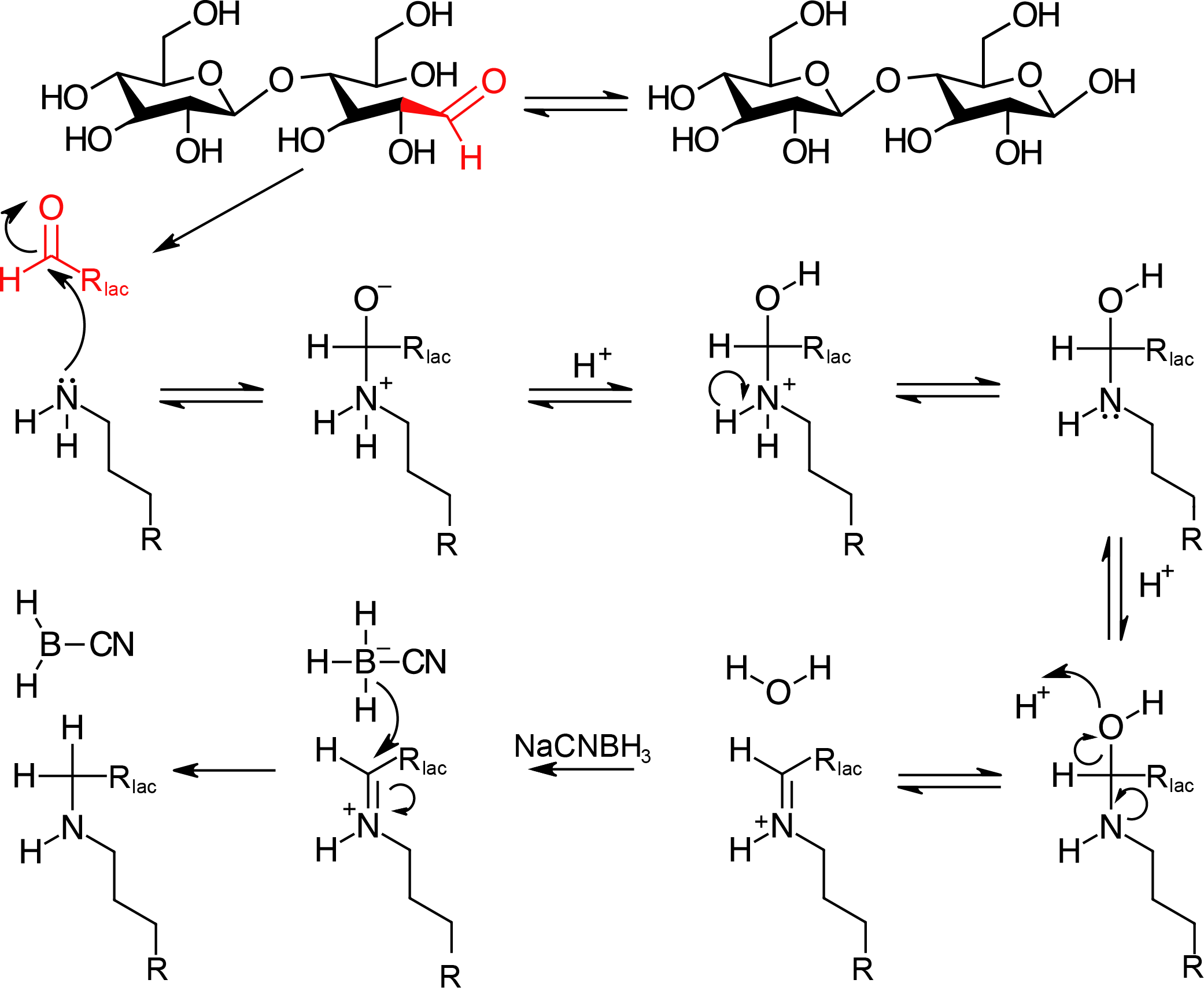


Fig. S3. Reductive amination mechanism for the reaction between lactose and a lysine side chain (modified from *Advanced Organic Chemistry 1990* [2]).

***Mass spectrometry analysis to determine the degree of antibody lactosamination***


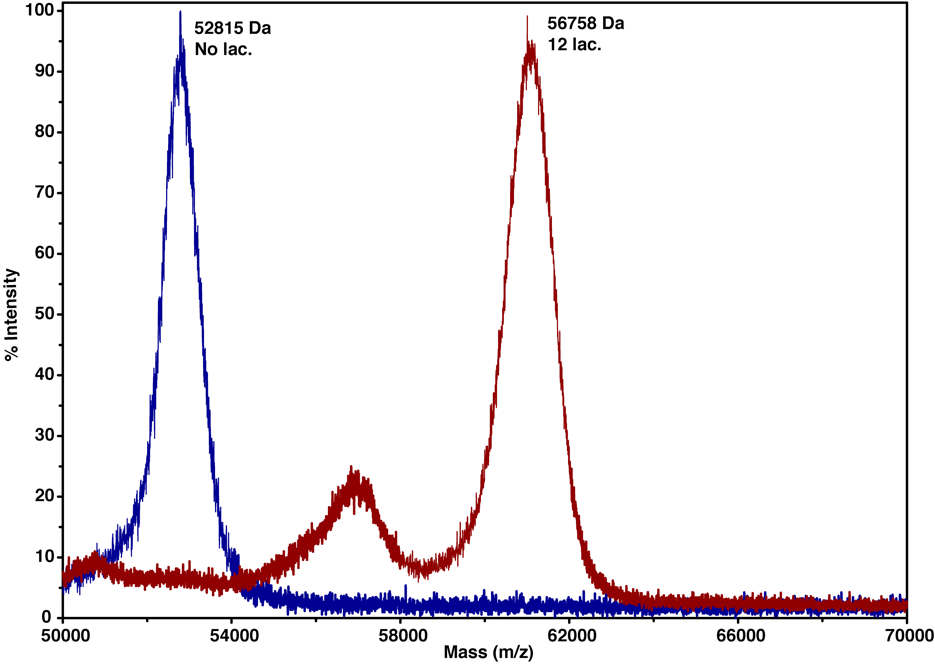


Fig. S4A) MALDI-MS spectra of unmodified (blue line) and lactosaminated (red line) cetuximab heavy chains. The individual peaks corresponding to different numbers of lactose units were not visible because of the lower resolution of the instrument at higher molecular weights, but a mass average was provided corresponding to 12 lactose units conjugated to each heavy chain.

**
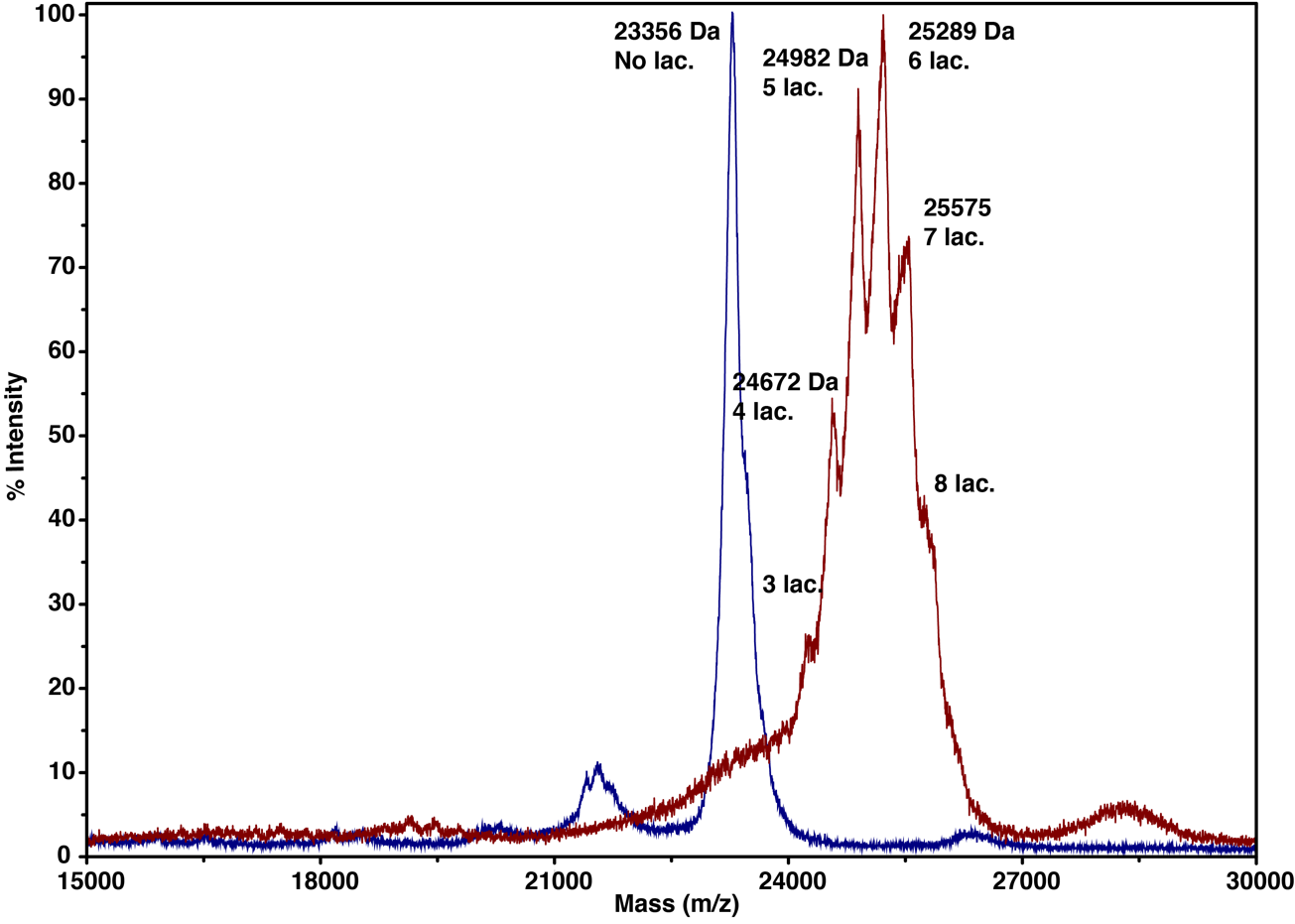
**

Fig. S4B) MALDI-MS spectra of unmodified (blue line) and lactosaminated (red line) cetuximab light chains. The peak corresponding to six lactose units was the most abundant. The average number of lactose units for each light chain was calculated to be 5.7.

Peak intensities relative to the most abundant peak:

3 lactose: 0.27

4 lactose: 0.55

5 lactose: 0.92

6 lactose: 1

7 lactose: 0.74

8 lactose: 0.43

***CA and T-ZHP1 concentration measurement by SDS-PAGE***

The concentration of CA and T-Z*HP1* was determined as described by Westerlund 2019 [3]. A series of known amounts of trastuzumab and cetuximab (0.32, 0.64, 0.96, 1.28, 1.6, 1.92 µg) was diluted in 20 mM citrate-phosphate buffer, 150 mM NaCl, pH 6. Both the standards and the samples where reduced with 50 mM DTT at 37 °C for 30 min and alkylated in the dark for 15 min with iodoacetamide. The standards and the samples where loaded onto an SDS-PAGE gel, which was run, stained and scanned (see Fig. S5). The intensity of the bands corresponding to the light chains on the scanned images was determined with the ImageJ software. The known samples where plotted and a linear equation was calculated by inserting the band intensities as numeric values in Excel for Mac 2011 (Microsoft). The linear equation was used to calculate the concentrations for the unknown samples of CA and T-*ZHP1*.

**
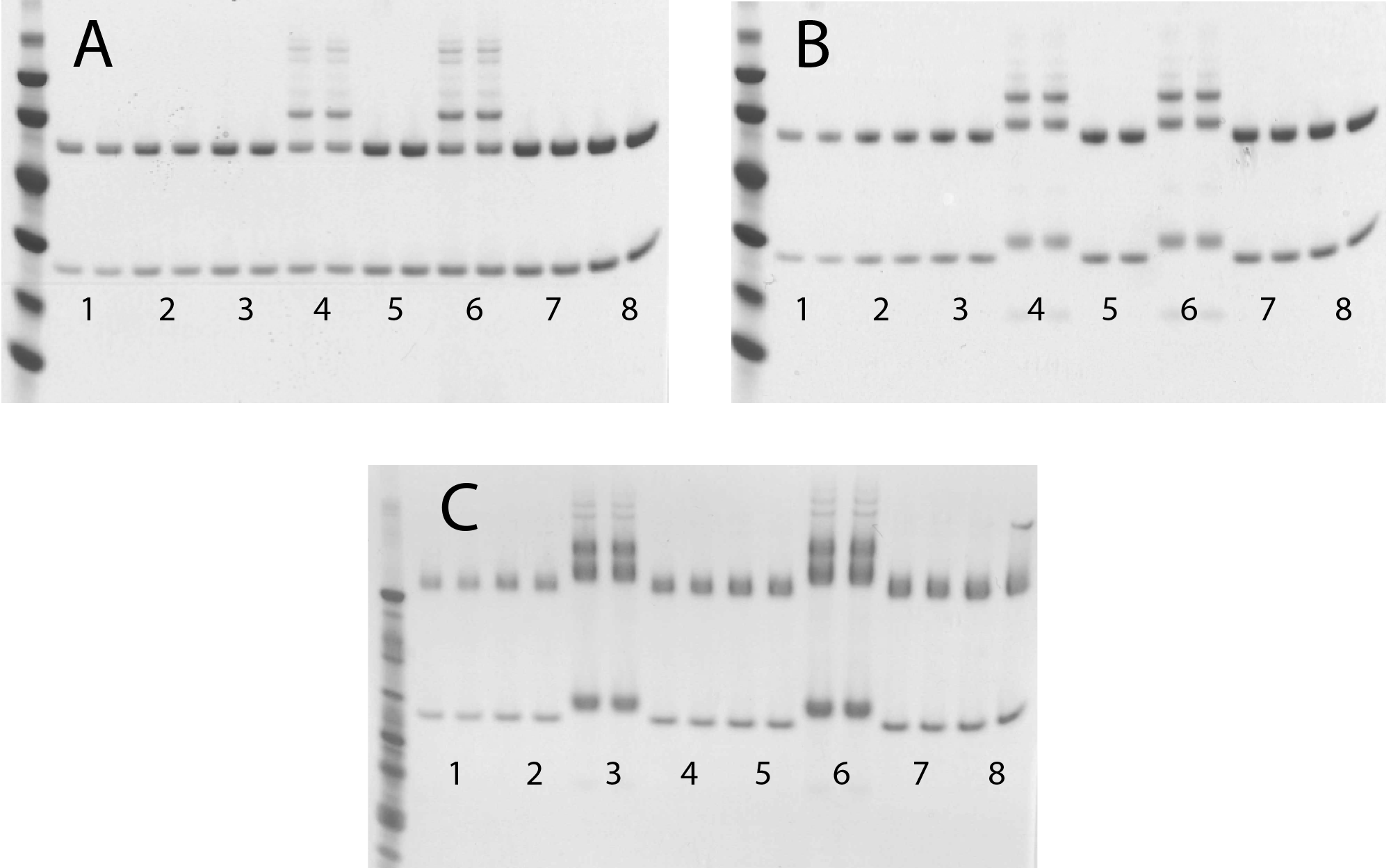
**

Fig. S5A) An unknown sample of T-Z*HP1* is loaded into lanes 4 and 6 in duplicates. Lanes 1-3, 5, and 7-8 correspond to different known amounts of unconjugated trastuzumab. Photoconjugation of Z*HP1* to the antibody gives rise to a new band, corresponding to the heavy chain covalently conjugated to Z*HP1*. B-C) An unknown sample of CA, produced by photoconjugation of Z_K35C-MBP_-*HP2’* (B) and Z_35BPA_-*HP2’* (C) to lactosaminated cetuximab, is loaded into lanes 4 and 6 in duplicates. Lanes 1-3, 5, and 7-8 correspond to different known amounts of unconjugated cetuximab. All bands are shifted towards higher molecular weights, which is explained by the added mass from the conjugated lactose units. It can be noted that the lactosamination gives rise to slightly blurred bands.

***Visualization of the binding interface between the B domain and IgG***
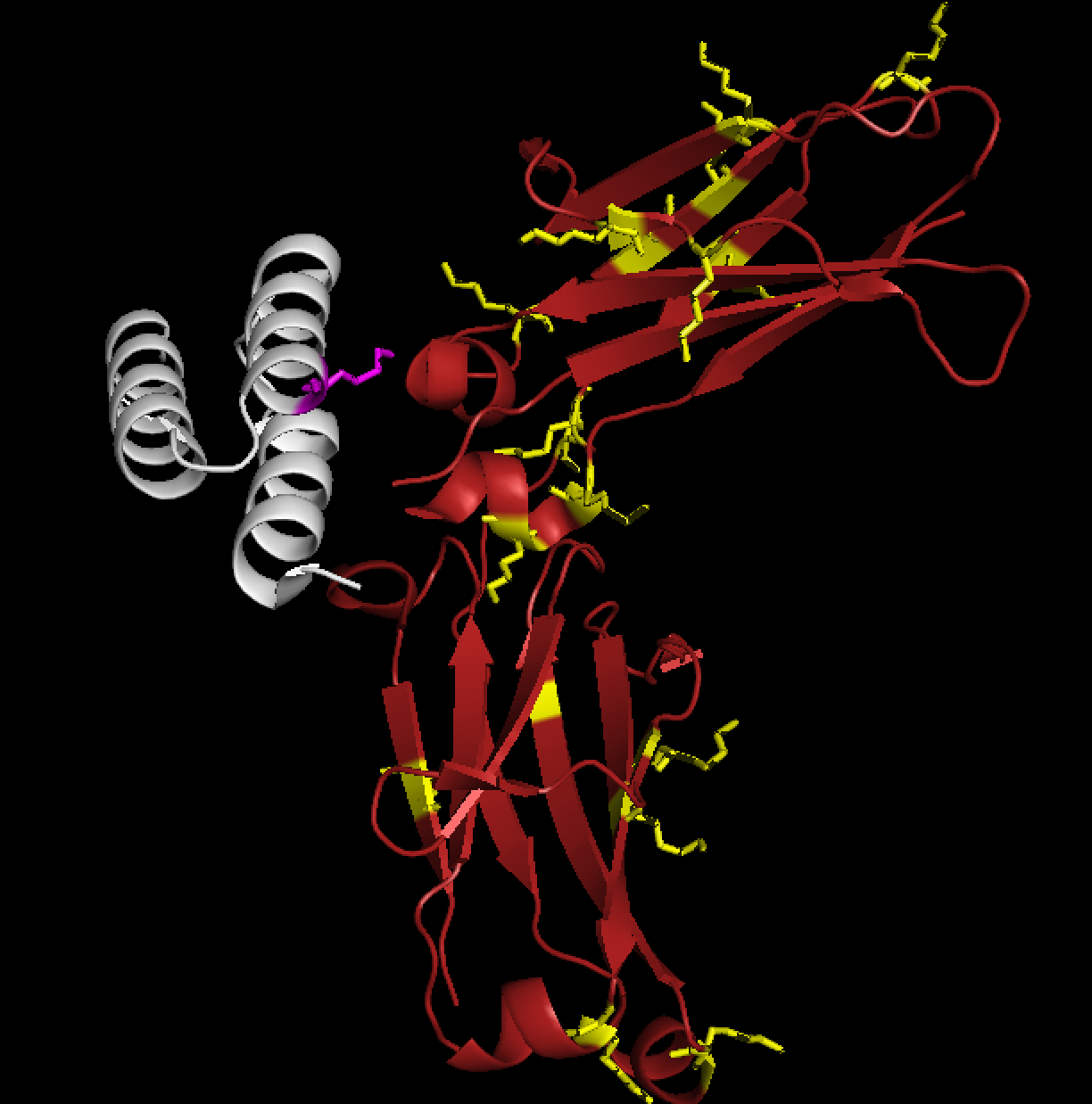


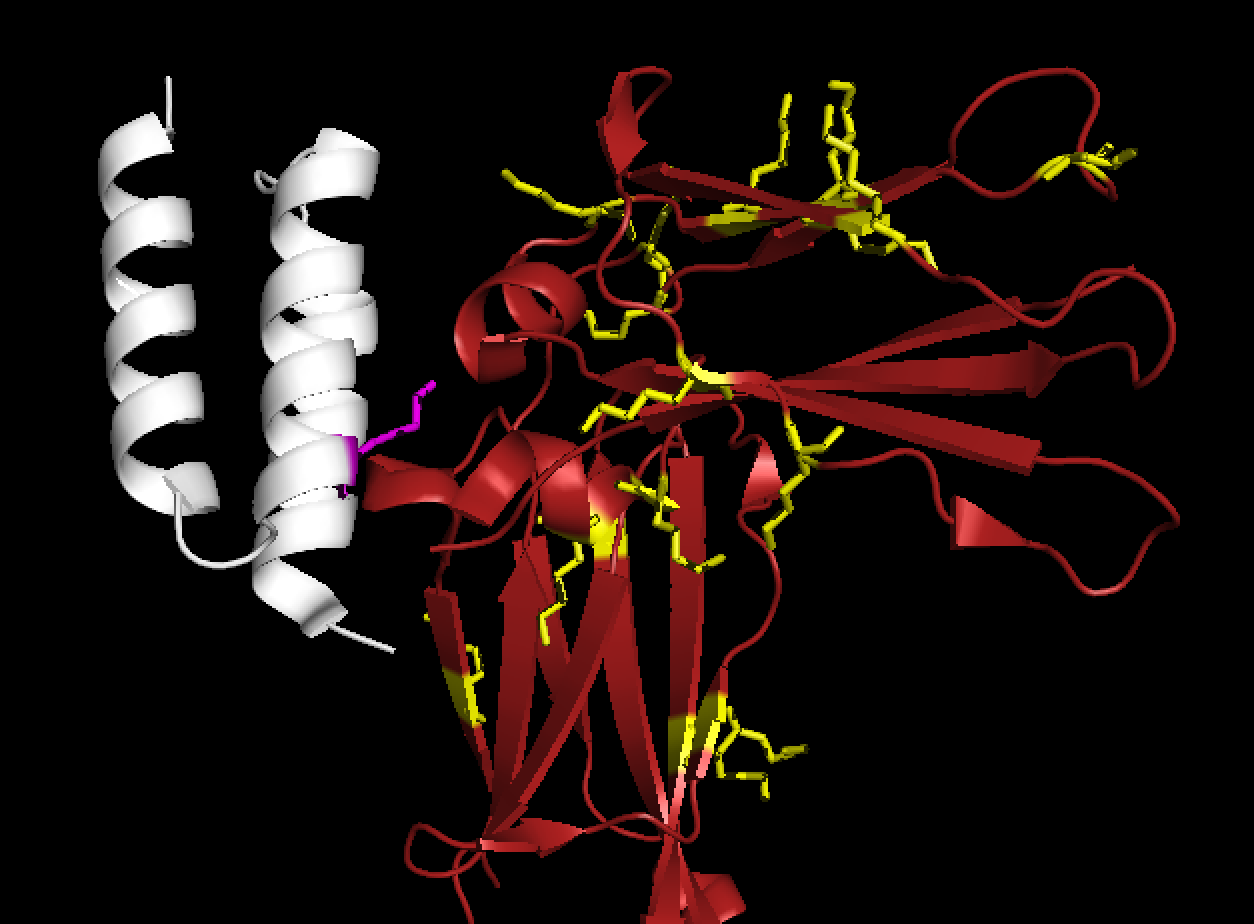


Fig. S6. Different views of a ribbon representation of the complex between the B domain (from which the Z domain is derived) and the Fc region of IgG (PDB file 5u4y). Lysine residues in IgG are shown in yellow. Position 35 in the B domain, used for incorporation of the benzophenone photoconjugation probe, is shown in pink.


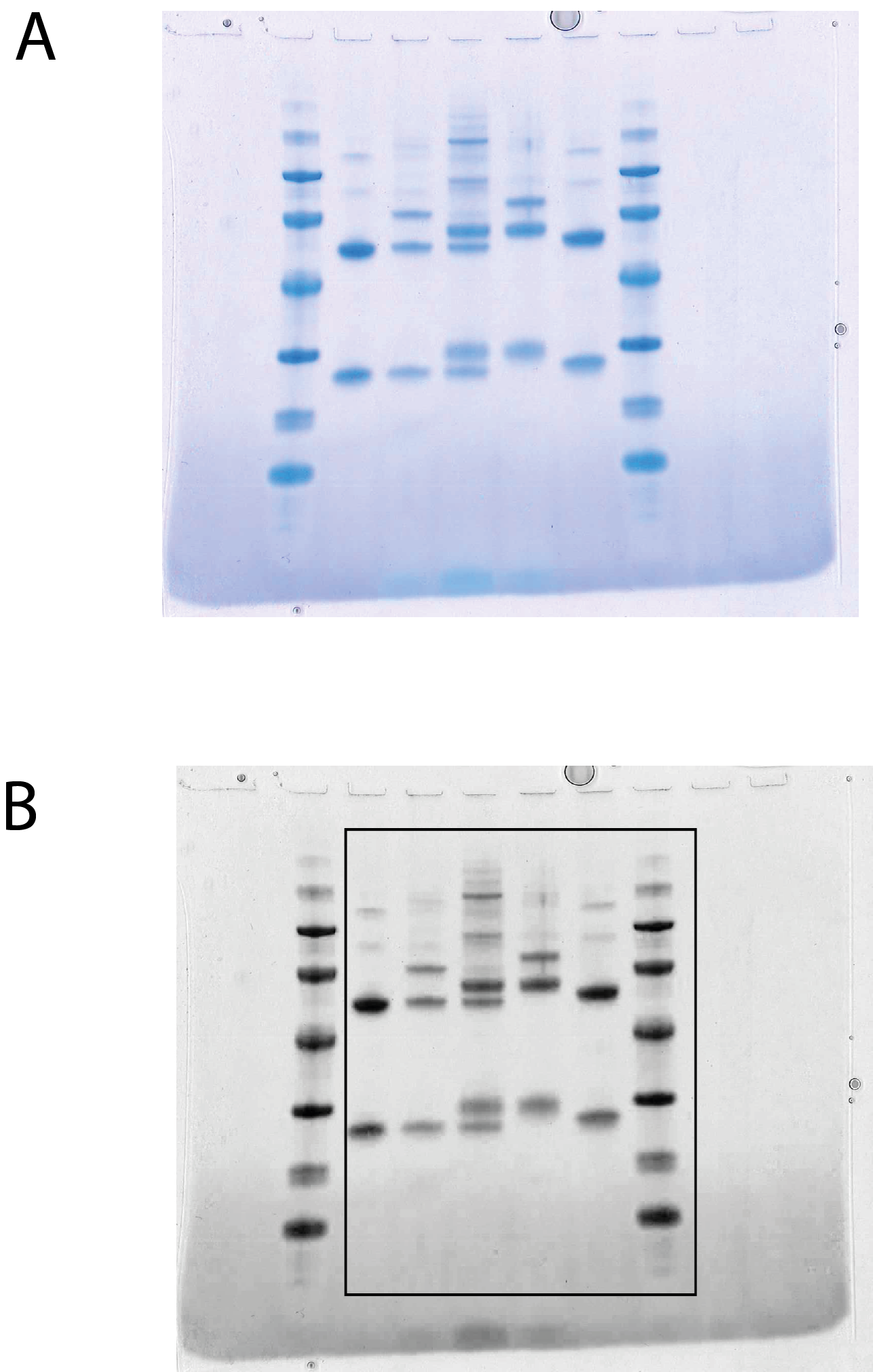


Fig. S7. Uncropped gels from Fig. 3. A) The original SDS-PAGE gel. B) The same gel as in A after black and white adjustment in Adobe Photoshop. The black frame indicates the area shown in Fig. 3A.

***References***

1. Knight, P. Hydrolysis of P-NN’-phenylenebismaleimide and Its Adducts with Cysteine. Implications for Cross-Linking of Proteins. *Biochem. J.* **1979**, *179* (1), 191–197.

2. Carey, F. A.; Sundberg, R. J. *Advanced Organic Chemistry: Part B: Reactions and Synthesis*; 1990.

3. Westerlund, K.; Vorobyeva, A.; Mitran, B.; Orlova, A.; Tolmachev, V.; Karlström, A. E.; Altai, M. Site-Specific Conjugation of Recognition Tags to Trastuzumab for Peptide Nucleic Acid-Mediated Radionuclide HER2 Pretargeting. *Biomaterials* **2019**, *203*, 73–85.
